# Avoiding causal fraud in the evaluation of clinical benefits of treatments for Alzheimer's disease

**DOI:** 10.1002/alz.14457

**Published:** 2025-01-27

**Authors:** Kathy Y. Liu, Stephen Senn, Robert Howard

**Affiliations:** ^1^ Division of Psychiatry University College London London UK; ^2^ Sheffield Centre for Health and Related Research (SCHARR) The University of Sheffield Sheffield UK

**Keywords:** Alzheimer's disease, clinically meaningful, minimal clinically important difference, responder analysis, trial, within‐individual change

## Abstract

Recent regulatory approvals of three amyloid‐lowering monoclonal antibody therapies for the treatment of Alzheimer's disease (AD) have triggered a polarizing debate in the field on the clinical meaningfulness of their reported effects. The question of how to define clinical meaningfulness for any treatment that has a modest effect size is important and will likely be subject to influence from interested stakeholders. We warn of claims of evaluating meaningful within‐individual change from randomized parallel‐group trials of AD treatments, sometimes purportedly assessed by a commonly recognized “responder” analysis approach, and explain why it is likely to mislead and should simply be avoided. The average between‐group difference in score change is where the debate and research efforts should be focused to contextualize and evaluate the clinical meaningfulness of the true treatment effect. The statistical and communication principles we consider and would recommend are applicable to the evaluation of most interventions in medicine.

**Highlights:**

- Dichotomized outcome analysis approaches purporting to evaluate within‐individual meaningful change are highly likely to mislead.
- In our view, the most valid statistical approach to understanding the true treatment effect is to analyze the average between‐group difference in outcome scores.
- The average between‐group difference in score change is where the debate and research efforts should be focused to contextualize and evaluate the clinical meaningfulness of the true treatment effect.

## 1 MAIN TEXT

There is no consensus on how to define clinical meaningfulness most accurately and informatively for treatment outcomes in Alzheimer's disease (AD) trials. Since the publication of outcomes data from pivotal licensing trials of aducanumab,^1^ lecanemab,^2^ and donanemab,^3^ three US Food and Drug Administration (FDA)‐approved amyloid‐lowering monoclonal antibody therapies for the treatment of AD,^4^, ^5^, ^6^ various approaches have been proposed to define and evaluate a clinically meaningful difference.^7^ Our view is that for parallel‐arm AD randomized controlled trials (RCTs) using a continuous outcome measure, the most valid statistical approach is to look at the average between‐group difference in (primary) outcome, which corresponds to the average variation between treatment and placebo groups^8^ and the true treatment effect above the placebo effect. Other indirectly derived approaches to present trial findings have serious statistical limitations or depend on as yet unsupported assumptions, for example, the “time saved with treatment” approach assumes a linear pattern of cognitive/functional decline in early AD stages, and the “percentage slowing” approach assumes the treatment has disease‐modification properties and will confer cumulative benefit.^7^

Another persistent approach has been to compare numbers or proportions of individuals in treatment and placebo groups who experienced a threshold‐defined level of clinically meaningful change to show that the treatment was associated with a higher likelihood of meaningful change within individuals. The FDA has encouraged a movement toward a “patient‐focused” approach, accompanied by an emphasis on establishing meaningful change in outcome measures at the individual level versus at the treatment group.^9^, ^10^ Suggested thresholds of within‐individual clinically meaningful change for AD include progression to the next category of a clinical staging instrument or empirically defined minimal clinically important difference (MCID) estimates. It is often argued, without clear statistical justification, that such thresholds are intended to define meaningful *change within individuals* and it is inappropriate to apply them to *between‐group mean differences*.^11^, ^12^, ^13^ While these are distinct concepts, we would emphasize that it is not possible to attribute individual outcomes observed in a parallel‐arm RCT to a treatment effect because this trial design identifies only *between‐treatment* and not *within‐individual* variation. For this reason, the suggested within‐individual change approach, often presented as a comparison of the proportion of individuals who experienced a threshold‐defined meaningful change between treatment and placebo groups, is highly likely to mislead and risk committing what we term “causal fraud.”

We use a simulated AD parallel‐arm RCT to show that observed between‐group differences in proportions of individuals who have achieved a certain binary outcome are derived from a treatment‐related shift in the distribution of scores, which cannot quantify the true treatment response within individuals. It is imperative that researchers, clinicians, sponsors, and regulators are clear about the statistical transformations involved in these within‐individual analyses and their limitations, as they can present trial findings in a more favorable light than the data support, and that can ultimately influence decisions made by patients and their families.

### 1.1 Average difference between groups

**FIGURE 1:**
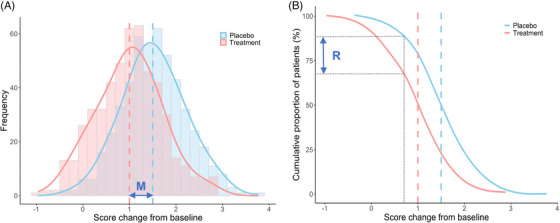
A simulated model of the normally distributed changes in score from baseline in a parallel‐arm RCT. Trial arms comprise treatment (red, *n* = 500, mean = 1 point, SD = 0.7) and placebo (blue, *n* = 500, mean = 1.5 points, SD = 0.7) groups. In panel (A), histogram bars display the frequency distribution of score changes and overlaying density curves illustrate the probability density function of score changes. In panel (B), CCDF curves show the proportion of individuals in each group who have achieved a score change from baseline equal to or greater than a given value on the *x* axis. Vertical dashed lines represent the mean changes in score from baseline for each group. Compared to placebo, the treatment shifts the score distribution and group mean to the left, and the *mean between‐group difference* in score change from baseline (M) is 0.5 points. In panel (B), a hypothetical clinically meaningful threshold of 0.75 points (dotted black line) is used to dichotomize score changes from baseline to define proportions of “responders,” or in the case of meaningful decline, “progressors,” who experienced a 0.75‐point score increase. These proportions can be compared between groups to give a difference in progressor risk or rate (R). Approximately 70% of the treatment group are defined as “progressors” versus 85% of the placebo group, which can be presented as a 15% risk (or 18% relative risk) reduction of experiencing clinically meaningful decline associated with treatment. This illustrates why the mean between‐group difference (particularly via MMRM approaches) provides a more accurate estimate of the true treatment effect compared to an alternative responder/progressor analysis. CCDF, complementary cumulative distribution function; MMRM, mixed models for repeated measures; RCT, randomized controlled trial; SD, standard deviation.

In a simulated parallel‐arm RCT, in which AD participants (*n* = 1000) are randomized to receive either a placebo or active drug, the change from baseline scores in each group are continuous measures. It is not essential to our argument, but it simplifies the discussion if we assume that these measures will be approximately normally distributed under placebo. In Figure 1A, we assume that the effect of treatment is to shift this distribution to the left. The basic logic of a randomized parallel‐group trial is that the placebo group can act as a proxy for what would have been seen for the treated patients had they been given a placebo instead. If the effect of treatment is to give the same constant benefit to every patient (compared to what their placebo value would have been), the figure represents what we would see. Of course, such constant shift is not the only possible explanation, but without further evidence, we cannot know. For the moment, we assume that such a simple shift is the case and consider the consequences.

In Figure 1A, the mean difference in score changes from baseline between treatment and placebo groups at the trial endpoint, a standard a priori primary outcome for AD treatment RCTs, is associated with a shift of the score distribution (to the left for treatments of AD in which all trial participants continue to decline and higher scores on the outcome scale represent worse cognitive/functional decline). Usually (and as was the case for aducanumab,^1^ lecanemab,^2^ and donanemab^3^ phase 3 RCTs), mean between‐group differences from longitudinal RCTs are analyzed using mixed models for repeated measures (MMRM) approaches, which can provide a more accurate estimate of the true treatment effect than a simple mean difference as they can account for multiple sources of variability. These include between‐individual variability by incorporating covariates such as trial group membership, baseline differences, use of medication, apolipoprotein E ε4 carrier status, and geographic region, as well as within‐individual variability by incorporating random effects over repeated measures. Although, if there are no missing data and the covariates can be treated as fixed, that is, having a constant effect on the outcome across all individuals, there is usually very little, if any, advantage to using a mixed model over simple summary measures approaches.^14^

Clinically, the average between‐group difference is a useful statistic because it is both accurate and straightforward to communicate to patients and their families that, on average, the size of the benefit (or the reduction in cognitive/functional decline), above effects of placebo, after taking the drug for a certain duration was equivalent to, for example, 0.5 points on an outcome scale. Even very small differences in score can become statistically significant with a large enough sample size,^15^ and it becomes more difficult to interpret and communicate the clinical relevance of smaller treatment effects, particularly those that are a fraction (e.g., less than half) of a point on a clinical symptom scale. In our view, the average between‐group score difference is where the debate and research efforts should be focused to contextualize and evaluate the clinical meaningfulness of the average true treatment effect, for example, whether it represents (for individuals or groups) a noticeable *and* valuable benefit that is worthwhile when weighed against risks, costs, and/or inconvenience of treatment.^7^

### 1.2 Dichotomization of continuous outcomes and pitfalls of responder analyses

A persistent argument, without clear statistical justification, has been that thresholds of clinical meaningful response can only be applied to within‐individual change,^11^, ^12^, ^13^ which involves dichotomizing a continuous outcome measure to obtain a binary outcome. For example, an MCID estimate is proposed as a threshold that can define individuals who experienced a (minimal) clinical meaningful change and those who did not. Statistical approaches that rely on a dichotomized binary outcome, including responder analyses (which can be transformed into numbers needed to treat) and time‐to‐event analyses, result in a loss of information and reduced statistical power, increasing the risk of false positives and negatives. The precise threshold chosen will directly influence the findings, and a single MCID threshold is unlikely to apply to all individuals in all contexts. For example, available empirical MCID estimates for AD comprise mean score changes anchored to clinician‐rated judgments of (minimal) meaningful change,^13^, ^16^ which can be influenced by duration of follow‐up (and degree of recall bias), cohort characteristics, and disease severity.

A “responder analysis” is a specific binary outcome analysis that presents and compares the proportion of individuals who surpass a threshold used to define a clinically meaningful “response” in each group, from which a (statistically significant) risk difference or relative risk associated with treatment can be calculated. Figure 1B shows the distribution of scores in treatment and placebo arms in a simulated RCT using complementary cumulative distribution functions, where the *y* axis shows the proportion of individuals in each group who have achieved a score change from baseline *equal to or greater than* a given value on the *x* axis. Empirical MCID estimates can define individuals who have experienced a clinically meaningful level of cognitive/functional worsening, that is, a “progressor” (which can be considered the inverse of a “responder”). In Figure 1B a hypothetical MCID threshold of 0.75 points defines a proportion of individuals who experienced a score change from baseline ≥ 0.75 points. In the simulated example, this is ≈ 75% in the treatment versus 90% in the placebo group, which can be presented as a risk difference of 15% and a relative risk reduction of 17% associated with treatment. For smaller “progressor” proportions, smaller risk differences can equate to larger relative risk reductions, for example, “progressor” proportions of 20% in the treatment versus 25% in the placebo group result in a 5% risk difference but a 25% reduction in relative risk. This is relevant as 18‐month RCTs of individuals with early AD, defined as mild cognitive impairment and mild AD dementia, can be expected to show lower overall proportions of “progressors” compared to longer trials or those conducted at more severe AD stages.

**FIGURE 2:**
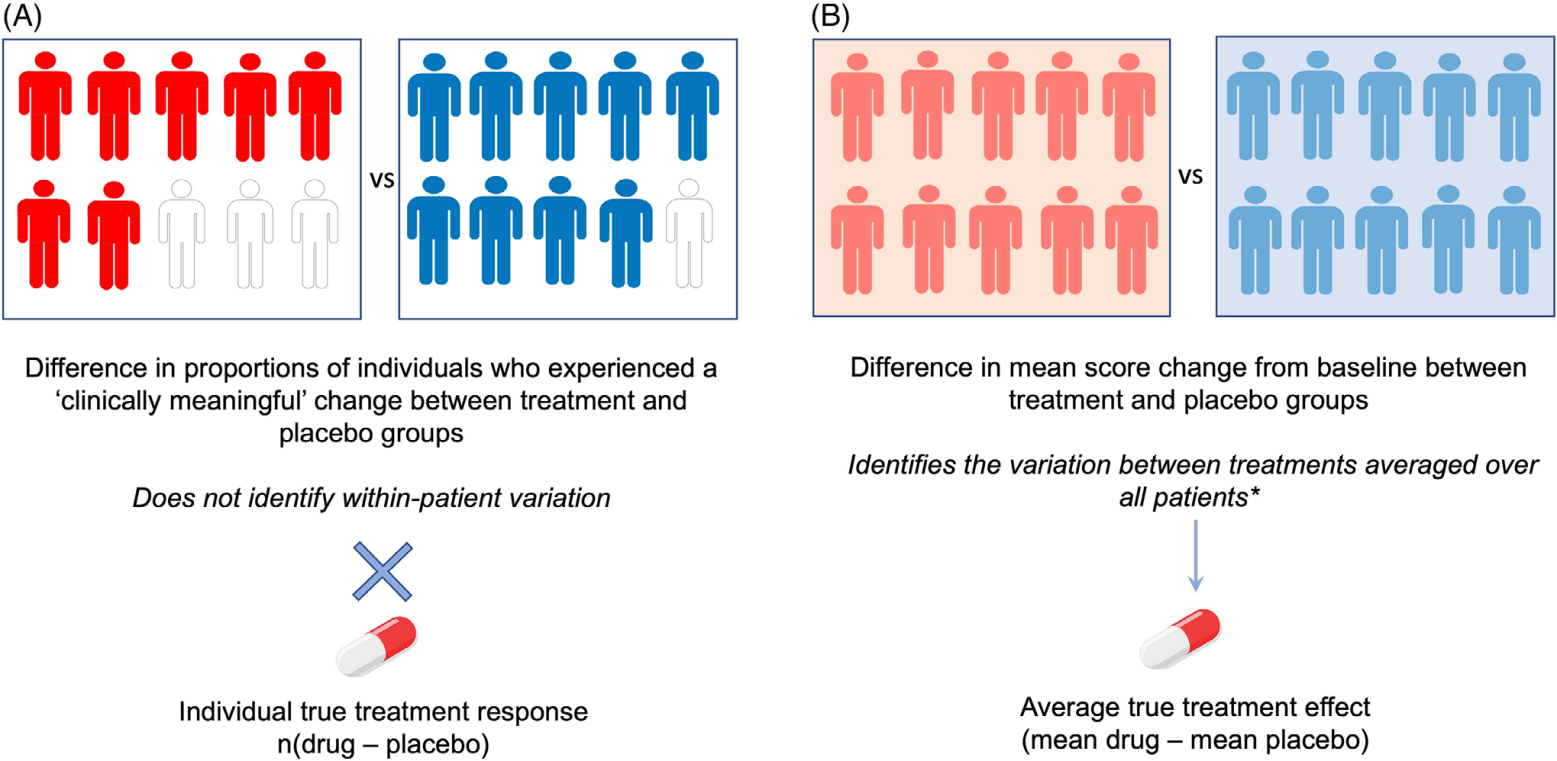
Comparison of two approaches to evaluate the treatment effect from parallel‐arm RCT data. A responder analysis (A) is proposed to evaluate within‐individual meaningful change by comparing the proportions of individuals who experienced a clinically meaningful change between treatment and placebo groups. However, this approach in a parallel‐arm RCT does not reflect the within‐individual variation (response) associated with a true treatment effect, which requires repeated crossover RCT or n‐of‐1 trial designs. In contrast, the average between‐group difference in score change from baseline (B) identifies the between‐treatments variation, which reflects the average variation attributable to a true treatment effect. *Mixed models for repeated measures approaches can account for some components of between‐individual variation (by incorporating covariates such as trial group, baseline differences, use of medication, apolipoprotein E ε4 carrier status, and geographic region) and within‐individual variation by incorporating random effects over repeated measures. RCT, randomized controlled trial.

While it is not inaccurate per se to report observed proportions in each group in a parallel‐arm RCT who have achieved a certain binary outcome, this approach is highly likely to mislead,^17^ as illustrated in Figure 2. Comparing responder rates between treatment and placebo groups can lead to the erroneous interpretation that the treatment increased the chance that a patient achieved a clinically meaningful benefit when, in fact, the parallel‐arm RCT design precludes the identification of within‐individual variation attributable to treatment. To put it another way, if all individuals have the same true shift there is no distinction between the mean effect for the group of patients and the effect for any given patient and arguing that the latter is the superior way to understand treatment effects is false. Furthermore, given measurement error, the mean group effect will provide a more accurate estimate. Conversely, if there is measurement error, the fact that observed within‐subject differences differ from subject to subject cannot, on its own, be taken as evidence that the true effect differs. A more careful analysis is required. The identification of an interaction (where the effect of the treatment varies depending on the presence or level of another factor) requires replication at the level at which the interaction occurred.^17^ Thus, for example, a treatment‐by‐sex interaction can be identified if there are sufficient males and sufficient females in the trial to compare the treatment effects between the sexes. However, in a parallel‐group trial there is no replication at the level of the individual.

In other words, it is impossible to know the extent to which the observed “response” was caused by the treatment; for that, we would need a repeated crossover RCT, or n‐of‐1 trial design in which patients are randomized to sequences in which they receive treatment and placebo at least once to evaluate within‐individual variation. At best, one might be able to judge that some variability in treatment effect has occurred if the spread of values differed markedly between groups. However, labeling patients as responders and non‐responders does not provide a valid shortcut to such inferences. It is entirely possible that any observed difference in responder rates is, in fact, due to a clinically trivial treatment effect (smaller than the dichotomization or MCID threshold of clinically meaningful change) that shifts a proportion of individuals who have a borderline “response” to placebo past the threshold to be classified as a responder. Responder analyses depend on the shift in the distribution of scores between groups but cannot quantify individuals’ true treatment response, let alone judge whether it was clinically meaningful. It is, therefore, inaccurate to attribute any difference in or risk reduction of response rates to a treatment effect. By comparison, the between‐group mean difference (via MMRM) provides a more accurate estimate of the (average) true treatment effect but stating that a treatment resulted in a 25% reduction in relative risk of clinically meaningful progression is likely to sound more favorable than saying it resulted in an average benefit equivalent to half a point on a cognitive scale, which might be smaller than empirical MCID estimates.^18^

## 2 CONCLUSION

Although we discuss here the situation for recently licensed treatments for AD, the statistical and communication principles we consider and would recommend are applicable to the evaluation of all interventions in medicine except possibly, when a true binary outcome, as opposed to a continuous one that might be dichotomized, is considered. Using a simulated AD parallel‐arm RCT, we demonstrate that there is more than one way to present a treatment‐related shift in the distribution of scores, but in our view, the most valid statistical approach to understanding the true treatment effect is to analyze the average between‐group difference in outcome scores. Persistent attempts to dismantle the validity of the average between‐group difference and replace it with dichotomized outcome analysis approaches purporting to evaluate within‐individual meaningful change, for example, responder proportions defined by a threshold of clinically meaningful change, are highly likely to mislead and risk committing “causal fraud” if any difference in responder proportions are presented as a treatment effect. Our view is that the debate should lie in whether the observed between‐group mean difference is clinically meaningful, and further work is needed to improve our understanding of how to interpret and communicate the clinical relevance of derived between‐group mean differences in trials as applied to diverse patient populations.

## CONFLICT OF INTEREST STATEMENT

K.L. and R.H. declare no competing interests. S.S. acts as a consultant to the pharmaceutical industry but is unaware of any conflict of interest; a full list of his interests is maintained here: http://senns.uk/Declaration_Interest.htm. Author disclosures are available in the Supporting Information.

## Supporting information

Supporting Information

## References

[1] Budd Haeberlein S , Aisen PS , Barkhof F , et al. Two randomized phase 3 studies of aducanumab in early Alzheimer's Disease. J Prev Alzheimers Dis. 2022;9:197‐210. 35542991 10.14283/jpad.2022.30

[2] van Dyck CH , Swanson CJ , Aisen P , et al. Lecanemab in early Alzheimer's Disease. N Engl J Med. 2022. doi:10.1056/NEJMoa2212948 36449413

[3] Sims JR , Zimmer JA , Evans CD , et al. Donanemab in early symptomatic Alzheimer Disease: the TRAILBLAZER‐ALZ 2 randomized clinical trial. JAMA. 2023;330:512‐527. 37459141 10.1001/jama.2023.13239 PMC10352931

[4] Center for Drug Evaluation, Research . FDA's decision to approve new treatment for Alzheimer's Disease. US Food and Drug Administration 2022. Accessed June 30, 2023. https://www.fda.gov/drugs/news‐events‐human‐drugs/fdas‐decision‐approve‐new‐treatment‐alzheimers‐disease

[5] Office of the Commissioner . FDA grants accelerated approval for Alzheimer's Disease Treatment. US Food and Drug Administration 2023. Accessed January 24, 2023). https://www.fda.gov/news‐events/press‐announcements/fda‐grants‐accelerated‐approval‐alzheimers‐disease‐treatment

[6] Center for Drug Evaluation, Research . FDA approves treatment for adults with Alzheimer's disease. US Food and Drug Administration 2024. Accessed July 25, 2024). https://www.fda.gov/drugs/news‐events‐human‐drugs/fda‐approves‐treatment‐adults‐alzheimers‐disease

[7] Liu KY , Walsh S , Brayne C , Merrick R , Richard E , Howard R . Evaluation of clinical benefits of treatments for Alzheimer's disease. Lancet Healthy Longev. 2023;4:e645‐651. 37924845 10.1016/S2666-7568(23)00193-9

[8] Senn S . Mastering variation: variance components and personalised medicine. Stat Med. 2016;35:966‐977. 26415869 10.1002/sim.6739 PMC5054923

[9] U.S Food and Drug Administration . Patient‐Focused Drug Development Public Workshop Guidance 4 Discussion Document 2019. Accessed April 19, 2021). https://www.fda.gov/media/132505/download

[10] US Food and Drug Administration . Guidance for industry. patient‐reported outcome measures: Use in medical product development to support labeling claims 2009. 10.1186/1477-7525-4-79 PMC1629006 17034633

[11] Petersen RC , Aisen PS , Andrews JS , et al. Expectations and clinical meaningfulness of randomized controlled trials. Alzheimers Dement. 2023; 1‐7. doi:10.1002/alz.12959 PMC11156248 36748826

[12] Van Dyck CH , O'Dell RS , Mecca AP . Disease severity and minimal clinically important differences in clinical outcome assessments for Alzheimer's disease clinical trials. Alzheimers Dement. 2023;9:e12388. 10.1002/trc2.12388 PMC10175943 37187779

[13] Lansdall CJ , McDougall F , Butler LM , et al. Establishing clinically meaningful change on outcome assessments frequently used in trials of mild cognitive impairment due to Alzheimer's Disease. J Prev Alzheimer's Dis. 2023; 10:9‐18. doi:10.14283/jpad.2022.102 36641605

[14] Senn S , Stevens L , Chaturvedi N . Repeated measures in clinical trials: simple strategies for analysis using summary measures. Stat Med. 2000;19:861‐877. 10734289 10.1002/(sici)1097-0258(20000330)19:6<861::aid-sim407>3.0.co;2-f

[15] Sullivan GM , Feinn R . Using effect size‐or why the P value is not enough. J Grad Med Educ. 2012;4:279‐282. 23997866 10.4300/JGME-D-12-00156.1 PMC3444174

[16] Andrews JS , Desai U , Kirson NY , Zichlin ML , Ball DE , Matthews BR . Disease severity and minimal clinically important differences in clinical outcome assessments for Alzheimer's disease clinical trials. Alzheimers Dement. 2019;5:354‐363. 10.1016/j.trci.2019.06.005 PMC6690415 31417957

[17] Senn S . Being efficient about efficacy estimation. Stat Biopharm Res. 2013;5:204‐210.

[18] Liu KY , Schneider LS , Howard R . The need to show minimum clinically important differences in Alzheimer's disease trials. Lancet Psychiatry. 2021;8(11):1013‐1016. doi:10.1016/S2215-0366(21)00197-8 34087114

